# Population structure of *Haemonchus contortus* from seven geographical regions in China, determined on the basis of microsatellite markers

**DOI:** 10.1186/s13071-016-1864-z

**Published:** 2016-11-15

**Authors:** Fangyuan Yin, Robin B. Gasser, Facai Li, Min Bao, Weiyi Huang, Fengcai Zou, Guanghui Zhao, Chunren Wang, Xin Yang, Yanqin Zhou, Junlong Zhao, Rui Fang, Min Hu

**Affiliations:** 1 State Key Laboratory of Agricultural Microbiology, College of Veterinary Medicine, Huazhong Agricultural University, Wuhan, 430070 Hubei Province People’s Republic of China; 2 State Key Laboratory of Veterinary Etiological Biology, Key Laboratory of Veterinary Parasitology of Gansu Province, Lanzhou Veterinary Research Institute, Chinese Academy of Agricultural Science, Lanzhou, 730046 Gansu Province People’s Republic of China; 3 Faculty of Veterinary and Agricultural Sciences, The University of Melbourne, Parkville, Victoria 3010 Australia; 4 College of Animal Sciences and Veterinary Medicine, Liaoling Medical College, Jingzhou, 121000 Liaoling Province People’s Republic of China; 5 Department of Veterinary Medicine, College of Animal Science and Technology, Guangxi University, Nanning, 530004 Guangxi Zhuang Nationality Autonomous Region People’s Republic of China; 6 College of Animal Science and Technology, Yunnan Agricultural University, Kunming, 650201 Yunnan Province People’s Republic of China; 7 College of Veterinary Medicine, Northwest A&F University, Yangling, 712100 Shaanxi Province People’s Republic of China; 8 College of Animal Science and Veterinary Medicine, Heilongjiang Bayi Agricultural University, Daqing, 163319 Heilongjiang Province People’s Republic of China

**Keywords:** *Haemonchus contortus*, Genetic diversity, Microsatellites, China

## Abstract

**Background:**

Studying genetic variation within and among *Haemonchus contortus* populations can inform some aspects of this parasite’s population genetics and epidemiology. However, almost nothing is known about such variation in China.

**Methods:**

Adult males of *H. contortus* (*n* = 184) representing seven distinct populations in China were collected, and genetic variation within and among these populations was explored using eight distinct microsatellite markers.

**Results:**

Genetic parameters, such as heterozygosity and inbreeding coefficient (*F*_*IS*_) indicated that all eight microsatellites were highly polymorphic. Various analyses (AMOVA, *F*_*ST*_, phylogenetic, structure, mantel test and population dynamics) revealed high within-population variation, low population genetic differentiation and high gene flow for *H. contortus* in China.

**Conclusions:**

This study provides a first snapshot of the genetic substructuring of *H. contortus* populations in China using polymorphic markers, and might provide a starting point for assessing genetic changes over space and time during or following the implementation of particular treatment or control strategies, or changes as a consequence of environmental, management and climatic factors.

**Electronic supplementary material:**

The online version of this article (doi:10.1186/s13071-016-1864-z) contains supplementary material, which is available to authorized users.

## Background

Studying genetic variation in parasitic worms has implications for studying their population genetics, epidemiology and evolution [1–3]. Various molecular methods have been used to explore such variation in some strongylid nematodes of socio-economic importance, including *Haemonchus contortus*, *Haemonchus placei*, *Ostertagia ostertagi* and *Teladorsagia circumcincta* [4–8]. Various population genetic studies of *H. contortus* in different countries (Australia, Brazil, China, Italy, Malaysia, Pakistan, the USA and Yemen) have been conducted using mitochondrial gene markers, such as those in the cytochrome *c* oxidase subunit 1 (*cox*1) and nicotine amide dehydrogenase subunit 4 (*nad*4) genes [5, 8–13]. These studies have revealed relatively high levels of genetic variability within populations and high gene flow among populations, particularly in geographical regions with ongoing host movement [5, 8, 11, 13]. Although mitochondrial markers are informative, they are maternally inherited, constraining analyses and interpretations somewhat [14] and, depending on the gene(s) used, are not always sufficiently variable to gain deep insights into population structures or substructures [2, 15]. Therefore, in some instances, polymorphic markers, including microsatellites, have been applied to various parasitic nematodes [6, 16–20]. Microsatellites are short, repetitive sequences, which are distributed at high prevalence and randomly in the genome [21], and are usually highly polymorphic and proposed to be selectively neutral [22].

Although microsatellite analyses have been applied specifically to *H. contortus* populations in Australia, some European countries (i.e. France, the Netherlands, Sweden and the UK) and, more recently, India [17–20], this is not the case for China. This is surprising, given that this nematode is likely the most economically important parasite of livestock in China [12]. Indeed, there is no detailed information on the population genetic structure(s) of *H. contortus* populations in this country using polymorphic markers. Therefore, in the present study, we investigated the structures of seven *H. contortus* populations from disparate geographical regions across China, including Inner Mongolia, using eight microsatellite markers.

## Methods

### *H. contortus* populations

A total of 184 individual adult males of *H. contortus* were collected from the abomasa of sheep or goats from seven geographical locations (23–29 specimens per location/population) in Heilongjiang and Liaoning in northeastern China; Shaanxi and Inner Mongolia in northwestern China; Hubei in central China; and Yunnan and Guangxi in southwestern China (Table 1; Additional file 1: Figure S1). These locations are in temperate to subtropical climate zones and are separated by distances of 800 to 5000 km (Additional file 1: Figure S1). Each individual adult male worm was identified morphologically to genus level [23], and specific identification was based on the sequence of the second internal transcribed spacer of nuclear ribosomal DNA (ITS-2) ([24]; GenBank accession no. X78803) (see below). Samples were stored in 70% ethanol and frozen at -20 °C until use.

**Table 1:** Population genetic data for eight microsatellite markers in seven populations of *Haemonchus contortus* from China. Deviations from Hardy-Weinberg equilibrium (significant deviations indicated by*, *P* < 0.002381, Bonferroni multiple tests correction) in seven *Haemonchus contortus* populations. Minus sign (–) indicates heterozygote deficiency, and a plus sign (+) indicates heterozygote excess

Population		Hcms15 (9^b^)	Hcms40 (7^b^)	Hcms28 (15^b^)	Hcms22co3 (8^b^)	Hcms19 (12^b^)	Hcms36 (10^b^)	Hcms33 (7^b^)	Hcms37 (6^b^)	All loci
Hubei (26^a^)	A	4	5	9	5	5	7	2	4	5.125
	*Ho*	0.680	0.462	0.769	0.231	0.423	0.769	0.423	0.923	0.585
	*He*	0.533	0.581	0.815	0.531	0.474	0.674	0.491	0.541	0.580
	*P*-value				–*				+*	
	*F*_*IS*_	-0.283	0.209	0.057	0.570	0.110	-0.144	0.141	-0.732	-0.009
Yunnan (26^a^)	A	4	5	9	4	6	5	5	4	5.250
	*Ho*	0.615	0.192	0.654	0.154	0.500	0.615	0.654	0.615	0.500
	*He*	0.535	0.619	0.810	0.512	0.501	0.636	0.627	0.548	0.598
	*P*-value		–*		–*					
	*F*_*IS*_	-0.153	0.694	0.196	0.704	0.002	0.034	-0.044	-0.127	0.167
Guangxi (24^a^)	A	4	6	11	4	4	5	2	3	4.875
	*Ho*	0.348	0.522	0.583	0.208	0.333	0.542	0.333	0.833	0.463
	*He*	0.390	0.708	0.831	0.490	0.332	0.660	0.507	0.529	0.556
	*P*-value			–*	–*				+*	
	*F*_*IS*_	0.111	0.268	0.302	0.580	-0.005	0.183	0.348	-0.594	0.171
Shaanxi (29^a^)	A	6	5	7	4	8	7	3	3	5.375
	*Ho*	0.483	0.414	0.655	0.276	0.793	0.552	0.414	0.897	0.560
	*He*	0.451	0.498	0.768	0.438	0.698	0.626	0.526	0.524	0.566
	*P*-value								+*	
	*F*_*IS*_	-0.073	0.171	0.149	0.374	-0.139	0.121	0.216	-0.731	0.010
Liaoning (23^a^)	A	4	3	10	4	7	5	2	3	4.750
	*Ho*	0.478	0.261	0.783	0.217	0.696	0.739	0.435	0.739	0.543
	*He*	0.428	0.649	0.832	0.467	0.724	0.635	0.502	0.532	0.596
	*P*-value		–*		–*					
	*F*_*IS*_	-0.120	0.604	0.060	0.540	0.040	-0.169	0.137	-0.401	0.090
Heilongjiang (27^a^)	A	5	4	10	3	7	7	3	4	5.375
	*Ho*	0.346	0.259	0.815	0.148	0.667	0.630	0.444	0.815	0.515
	*He*	0.602	0.635	0.808	0.237	0.675	0.727	0.514	0.532	0.593
	*P*-value	–*	–*							
	*F*_*IS*_	0.430	0.596	-0.009	0.379	0.013	0.136	0.137	-0.509	0.133
Inner Mongolia (29^a^)	A	4	3	10	5	7	5	4	2	5
	*Ho*	0.655	0.345	0.862	0.103	0.621	0.690	0.517	0.793	0.573
	*He*	0.581	0.503	0.848	0.731	0.707	0.667	0.584	0.506	0.641
	*P*-value				–*					
	*F*_*IS*_	-0.130	0.318	-0.017	0.861	0.123	-0.034	0.116	-0.582	0.107

^a^Total number of individuals for each population

^b^Total number of alleles for each marker across all populations

*Abbreviations*: *Hcms Haemonchus contortus* microsatellite, *A* number of alleles, *H*_*o*_ observed heterozygosity, *H*_*e*_ expected heterozygosity, *F*_*IS*_ inbreeding coefficient

### Isolation of genomic DNA

Genomic DNA was extracted from single worms using sodium dodecyl-sulfate/proteinase K treatment, followed by spin-column purification (Wizard DNA Clean-Up, Promega, Madison, USA) [25] and then stored at -20 °C until use.

### PCR amplification of ITS-2 and sequencing

To confirm the identity of the nematodes as *H. contortus*, the ITS-2 of each individual worm was amplified using the primers NC1 (5′-ACG TCT GGT TCA GGG TTG TT-3′) and NC2 (5′-TTA GTT TCT TTT CCT CCG CT-3′) [24]. Briefly, PCR was performed in 25 μl containing 10 mM Tris–HCl (pH 8.3), 50 mM KCl, 4 mM MgCl_2_,250 μM of each dNTP, 100 pmol of each primer, 1 U *Taq* DNA polymerase [TaKaRa, Dalian, China]) and 30–50 ng of DNA template (except for no-template controls). The cycling protocol was: initial denaturation at 94 °C for 5 min, followed by 30 cycles of denaturation at 94 °C for 30 s, annealing at 55 °C for 30 s and extension at 72 °C for 1 min, with a final extension of 72 °C for 5 min. Amplicons were examined on agarose gels (1.5%) to verify that they represented single bands and then column-purified (PCR-Preps, Promega) and sequenced directly (using primer NC2) in an automated DNA sequencer (ABI 3730).

### PCR amplification of microsatellite loci

Eight microsatellite markers (designated Hcms 15, 19, 22co3, 28, 33, 36, 37 and 40) were selected based on previous publications [17, 18]. PCR was performed in 20 μl containing 10 mM Tris-HCl (pH 8.3), 50 mM KCl, 4 mM MgCl_2_, 250 μM of each dNTP, 100 pmol of each primer, 1 U of *Taq* DNA polymerase (TaKaRa) and 30–50 ng of DNA template (except for no-template controls). The cycling protocol was: initial denaturation at 94 °C for 5 min, followed by 30 cycles of denaturation at 94 °C for 30 s, annealing at 50–60 °C (temperatures for individual primer sets, see refs. [17, 18]) for 30 s and extension at 72 °C for 1 min, with a final extension of 72 °C for 5 min. Each forward primer was labelled at their 5-ends with the fluorescent dye FAM (Hcms 22co3, 28, 33 and 37) or HEX (Hcms 15, 19, 36 and 40). The sizes of individual amplicons were established by capillary electrophoresis in an ABI Prism 3730 XL analyzer. Individual chromatograms were assessed using GeneMarker 1.51 software (Applied Biosystems, USA) to assign allele fragment lengths to individual samples. The data set was assessed for errors using the Micro-Checker program [26], and any errors were corrected.

### Data analyses

Genetic characteristics were estimated by calculating observed and expected heterozygosities (*H*_*o*_ and *H*_*e*_), the number of alleles (A) and population inbreeding coefficients (*F*_*IS*_) for individual loci using the program GenALEx [27]. Exact tests for deviation from Hardy-Weinberg equilibrium and pairwise linkage disequilibrium were conducted using Genepop [28]. To correct for multiple tests, Bonferroni corrections were employed to adjust significance values [29].

Pairwise *F*_*ST*_ values were calculated using the program Arlequin 3.5 [30] to evaluate the genetic differentiation among populations. Analysis of molecular variance (AMOVA) was used to estimate genetic diversity within and among populations using the same program (Arlequin 3.5). Genetic structure was inferred using Bayesian Markov Chain Monte Carlo (MCMC) approach, and the Bayesian-based analysis was run in the program STRUCTURE [31]. Principal coordinate analysis (PCoA) was performed using GenALEx software, preserving individual worm genotypes to plot individuals [27]. To determine the correlation of geographical distance and genetic distance, the Mantel test was conducted using the TFPGA package [32]. The Bottleneck program [33] was used to assess any possible, recent reduction in effective population size.

## Results

### Genetic diversity and Hardy-Weinberg equilibrium

All 184 adult specimens of *Haemonchus* were identified individually as *H. contortus* based on their ITS-2 sequence. Individual adult male worms from each of the seven populations were then genetically characterized using the eight microsatellite markers (Hcms 15, 19, 22co3, 28, 33, 36, 37 and 40). All of these markers displayed a high degree of polymorphism, with the number of ‘alleles’ per locus ranging from 6 to 15 (Table 1). The observed heterozygosity (*H*_*o*_) ranged from 0.103 to 0.923, and the expected heterozygosity (*H*_*e*_) from 0.237 to 0.848 (Table 1). For each population, the average number of alleles at all loci ranged from 4.750 to 5.375, and the average, expected heterozygosity for all loci was > 0.5 for all populations (Table 1). Among all seven populations of *H. contortus*, those from Shaanxi and Heilongjiang were most polymorphic, with an average number of 5.375 alleles, whereas the population from Liaoning had the smallest mean number of alleles (4.750).

Population genetic analysis on departure from Hardy-Weinberg equilibrium (HWE) was undertaken to assess the suitability of the eight selected markers to assess genetic variability in the seven *H. contortus* populations. For some marker/population combinations, there was no significant departure from HWE after sequential Bonferroni correction, and *F*_*IS*_ values were low (see Table 1). However, for each of five markers (Hcms15, Hcms40, Hcms28, Hcms22co3 and Hcms37), there was a significant departure from HWE in one to five populations with heterozygotic deficiency or excess (see Table 1). In the populations with heterozygosity deficiency, the observed heterozygosity (*H*_*o*_) was less than the expected heterozygosity (*H*_*e*_), and *F*_*IS*_ values were high (see Table 1), suggesting the presence of null alleles at four loci (Hcms15, Hcms40, Hcms28 and Hcms22co3). There was also evidence that null alleles were observed for the same four loci (assessed using Micro-Checker software; data not shown). Although there were deviations from HWE for five markers in some *H. contortus* populations (Hcms15/Heilongjiang; Hcms40/Yunnan/Liaoning/Heilongjiang; Hcms28/Guangxi; Hcms22co3/Hubei/Yunnan/Guangxi/Liaoning/Inner Mongolia; Hcms37/Hubei/Guangxi/Shaanxi; Table 1), there was no evidence to support linkage disequilibrium for any combination of loci for individual populations, suggesting that these loci were not genetically linked and could be used independently.

### Genetic structure

Pairwise *F*_*ST*_ values were calculated to estimate the degree of genetic differentiation among the seven *H. contortus* populations using the eight microsatellite markers (Table 2). Although most pairwise *F*_*ST*_ values were low (< 0.05), ranging from 0.0024 to 0.0640, the population from Inner Mongolia showed the highest level of genetic divergence from other populations, with *F*_*ST*_ values ranging from 0.0352 to 0.0640 (Table 2). Low *F*_*ST*_ values indicated limited differentiation and high gene flow among most of the populations.

**Table 2:** Pairwise *F*_*ST*_ values among seven *Haemonchus contortus* populations upon pairwise comparison

Population	Hubei	Yunnan	Shaanxi	Liaoning	Heilongjiang	Inner Mongolia	Guangxi
Hubei							
Yunnan	0.0055						
Shaanxi	0.0122	0.0421					
Liaoning	0.0159	0.0286	0.0120				
Heilongjiang	0.0238	0.0398	0.0030	0.0148			
Inner Mongolia	0.0352	0.0640	0.0478	0.0454	0.0603		
Guangxi	0.0024	0.0036	0.0328	0.0104	0.0331	0.0628	

**Fig. 1:**
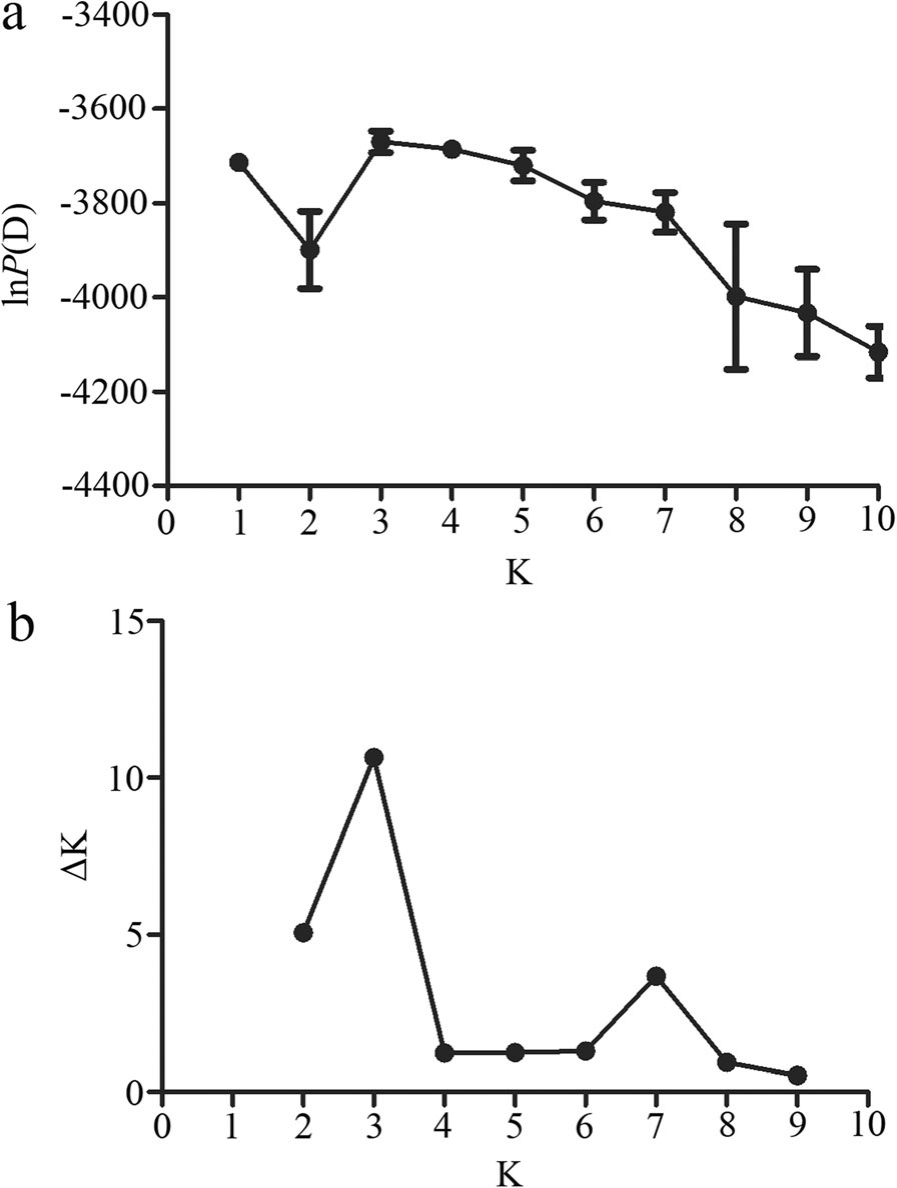
Genetic sub-structuring of *Haemonchus contortus* populations in China based on Bayesian cluster analysis. **a** Variation trend of ln*P*(D) values. ln*P*(D) was obtained by first computing the log-likelihood of the data in the program STRUCTURE. K values represent the number of clusters, and the *vertical bars* represent standard deviations. **b** Variation trend of ΔK values; ΔK is a predictor of the real number of clusters

Bayesian clustering analysis showed no obvious subdivision at the population level (results not shown). We explored K values between 1 and 10. For *K* = 3, corresponding ln*P*(D) was greatest, and the peak of ΔK was highest (see Fig. 1). To determine whether the *H. contortus* populations comprised a single panmictic population with a high degree of gene flow, an analysis of molecular variance (AMOVA) among populations was conducted (Table 3). An analysis using the STRUCTURE program supported the division of *H. contortus* populations into three groups: group 1 (Guangxi, Hubei and Yunnan), group 2 (Heilongjiang, Liaoning and Shaanxi) and group 3 (Inner Mongolia). Most (96.1%) of the genetic variation was distributed within populations: 3.3% of variance components among groups (*F*_*CT*_) and 0.6% of variance components among populations within groups (*F*_*SC*_). These findings suggest a very low genetic sub-structuring of *H. contortus* populations in China.

**Table 3:** Analysis of molecular variance (AMOVA) of *Haemonchus contortus* using eight microsatellite loci

Variance component	Variance	% of total	*F*-statistic	*P*-value
Among groups^a^	0.080	3.29	*F*_*CT*_ = 0.033	< 0.0001*
Among populations within groups	0.015	0.60	*F*_*SC*_ = 0.006	0.092
Within populations	2.363	96.11	*F*_*ST*_ = 0.039	< 0.0001*

^a^Seven populations were divided into three groups according to structure analysis, including (Hubei, Yunnan, Guangxi), (Shaanxi, Liaoning, Heilongjiang) and (Inner Mongolia)

*Significant effects (*P* < 0.0001)

### Geographical sub-structuring

**Fig. 2:**
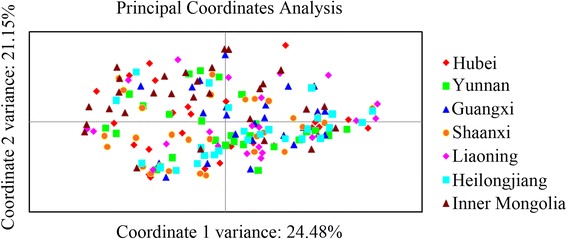
Principal coordinate analysis (PCoA) of individual genotypes representing seven populations of *Haemonchus contortus* in China using eight microsatellite loci. Each data point represents an individual worm. Each population is indicated by a different colour

Evidence of sub-structuring was assessed by Principal coordinate analysis (PCoA) analysis (Fig. 2). The two axes (i.e. 21.2 + 24.5%) accounted for 45.7% of the variation; the samples from different geographical origins were not distinct and formed an overlapping cluster, suggesting that there was no geographical sub-structuring.

**Fig. 3:**
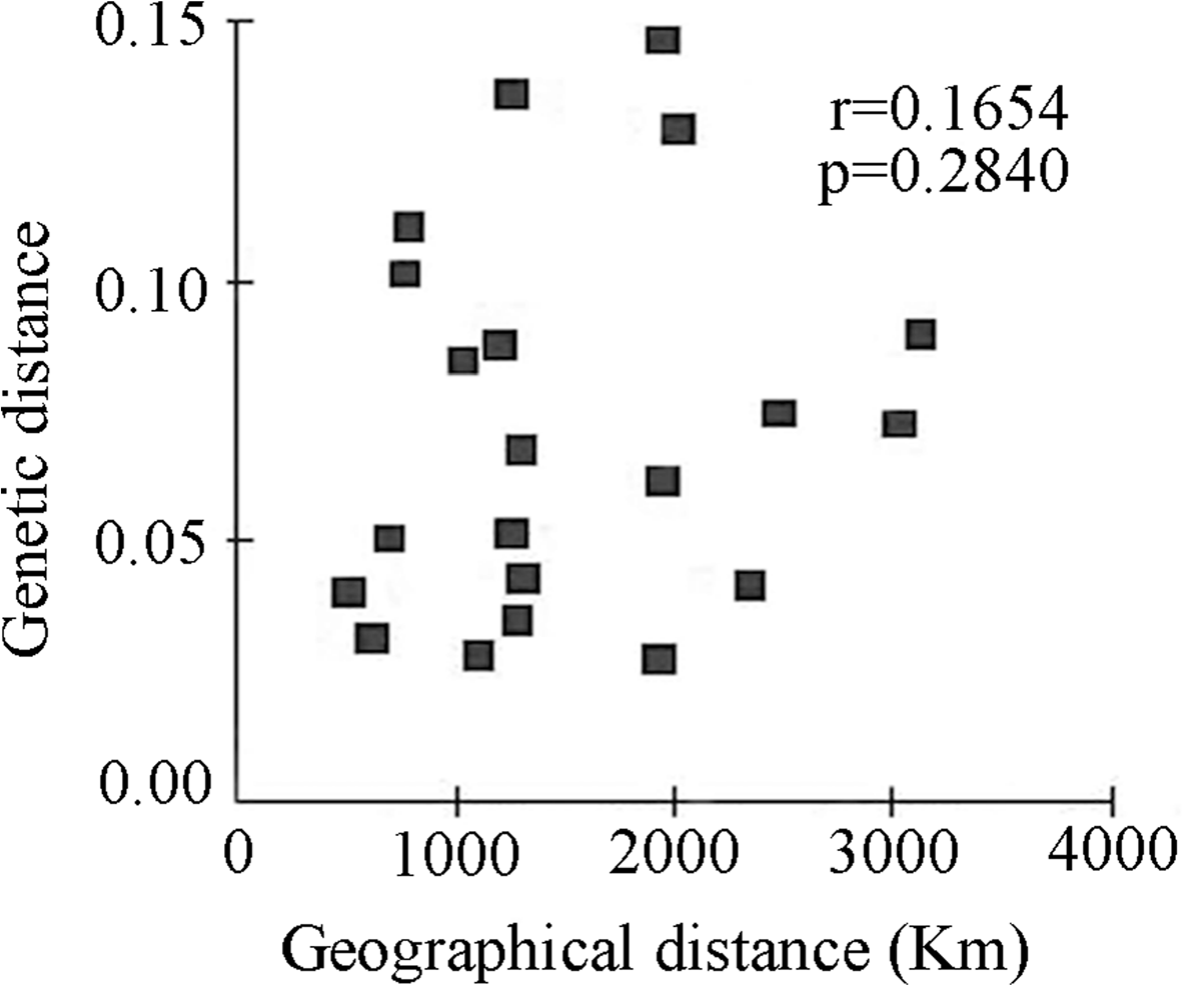
Nei’s genetic distance plotted as a function of linear geographical distance among seven populations of *Haemonchus contortus* in China

Nei’s genetic distance [34] was calculated to evaluate whether there was an effect of geographical separation on population differentiation. The analysis of the relationship between genetic distance and geographical distance for the seven *H. contortus* populations indicated that the genetic differentiation of these populations did not follow a pattern of isolation by distance (Mantel test: *r* = 0.1654, *P* = 0.284) (Fig. 3), indicating that genetic differentiation was not correlated with geographical distance for all populations, as a consequence of high gene flow among populations.

### Population dynamics

The analysis of the seven *H. contortus* populations showed that the populations from Inner Mongolia and Yunnan exhibited heterozygosity-excess, according to the IAM and TPM/SMM models of Sign test and the Wilcoxon signed-rank test (Table 4). The Mode-shift analysis demonstrated that the allele frequencies had a normal L-shaped distribution. Although the heterozygosity-excess was detected in two populations (i.e. Inner Mongolia and Yunnan), the results indicated that the populations studied did not appear to deviate from a mutation-drift equilibrium (Table 4).

**Table 4:** Bottleneck analyses of seven populations of *Haemonchus contortus* using eight microsatellite loci

Population	Sign test			Wilcoxon signed-rank test			Mode-shift test
	IAM	TPM	SMM	IAM	TPM	SMM	
Hubei	0.516	0.012	0.011	0.313	0.074	0.039	normal L-shaped
Yunnan	0.089	0.000*	0.000*	0.195	0.004*	0.004*	normal L-shaped
Guangxi	0.232	0.065	0.066	0.383	0.250	0.055	normal L-shaped
Shaanxi	0.218	0.189	0.052	1.000	0.055	0.039	normal L-shaped
Liaoning	0.226	0.208	0.200	0.027	0.945	0.461	normal L-shaped
Heilongjiang	0.259	0.054	0.054	0.074	0.039	0.019	normal L-shaped
Inner Mongolia	0.009*	0.080	0.210	0.004*	0.055	0.945	normal L-shaped

*Significant effects (*P* < 0.01, indicating heterozygote excess)

## Discussion

In the present study, we explored the population genetic structure of seven *H. contortus* isolates from China using a panel of eight polymorphic microsatellite markers. The allelic richness and overall heterozygosity of these microsatellites were shown to be high, supporting previous findings using mtDNA (*nad*4) nucleotide sequence data [12] and revealing high levels of genetic diversity within and among *H. contortus* populations in China.

Deviation from HWE was observed for five of eight markers for some populations (Hcms15/Heilongjiang; Hcms40/Yunnan/Liaoning/Heilongjiang; Hcms28/Guangxi; Hcms22co3/Hubei/Yunnan/Guangxi/Liaoning/Heilongjiang; Hcms37/Hubei/Guangxi/Shaanxi) from China, following Bonferroni correction, which is likely due to the presence of null alleles. As the repeated “genotyping” from the same samples gave consistent results, and individual samples that failed to amplify for one locus could be genotyped robustly for other loci, the null alleles might not relate to allelic drop-out or poor template quality; otherwise, the apparent ‘null alleles’ might relate to sequence variation at primer annealing sites and/or due to selection, mutation or genetic drift [7]. The high frequency of null alleles in Chinese *H. contortus* populations is in accordance with the previous studies of *H. contortus* [18] and other trichostrongylid nematodes including *Teladorsagia circumcincta* and *T. tenuis* [6, 7, 35].

No linkage disequilibrium was detected across the entire population. It is proposed that linkage disequilibrium results from a low level of genetic exchange, population sub-structuring, recombination and/or inbreeding [36]. The absence of linkage disequilibrium from Chinese populations of *H. contortus* was supported by AMOVA analysis results, which showed that the majority (96.1%) of genetic variation was within *H. contortus* populations, suggesting a high level of gene flow among populations, and PCoA results indicated that no discernable geographical sub-structuring was evident over large distances in China (Fig. 2). Therefore, both AMOVA and PCoA results demonstrated high gene flow and low geographical sub-structuring among the seven Chinese populations of *H. contortus*. The high rate of gene flow appears to relate to the frequent trade and transport of the sheep and goats with *H. contortus* infection across vast distances in China (cf. [12, 37]).

Although the numbers of samples studied here were relatively small, genetic differentiation between *H. contortus* populations from sheep and goats were not apparent, showing that there is no reproductive isolation [13]. Compared with other *H. contortus* populations in China, however, the population from Inner Mongolia showed a higher *F*_*ST*_ value (Table 2), which might relate to factors including unique local agricultural management and cultural factors in the Inner Mongolia autonomous region, the second largest plateau in China. The nomadic life style of people and animals and the economic isolation of Mongolia, and limited trade and communication with other regions, would explain limited livestock movement to other parts of China. Furthermore, confounding environment differences, such as climate, farm management and/or host factors (e.g. breed) appear to reflect particular epidemiological patterns of *H. contortus*.

In subtropical areas (Guangxi, Hubei and Yunnan) of China, the main climatic characteristics are warm temperatures and high rainfall in summer. Otherwise, winters are cold (-20 to -30 °C), and summers are hot (20 to 30 °C) and dry, with limited rainfall throughout much of the year in temperate zones (Heilongjiang, Liaoning and Inner Mongolia). Previous studies [38, 39] have shown that temperature and moisture have a dominant effect on the development and survival of the free-living stages of *H. contortus*; hence, the availability of infective larvae and rates of infection are affected. For this parasite, abundant rainfall and warm temperatures are favourable for development, but hot, dry and extreme cold conditions are usually lethal to infective larvae on pastures [40]. Consequently, the larval stages of *H. contortus* are often poorly adapted to cooler and drier climates, such that the perpetuation of the life-cycle is largely linked to the survival of parasitic stages in host animals to carry infection through from year to year [41]. The Autonomous Region of Inner Mongolia has a monsoon climate at medium latitudes, but temperature and precipitation appear to have changed in recent years, leading to a drier climate [42]. The ecological system in Inner Mongolia might limit the opportunity for the transmission as well as recombination, dispersal and genetic exchange of *H. contortus* with other populations in China. Thus, the low level of genetic exchange and higher genetic diversity in *H. contortus* might be impacted by environmental and/or microclimatic factors in Inner Mongolia.

The results of the low level of genetic differentiation among the seven *H. contortus* populations in China by microsatellite analysis were essentially in agreement with those of a previous study based on mitochondrial *nad*4 gene sequence data [12]. These findings are also supported by the proposal that limited genetic structuring of *H. contortus* populations might be ascribed to a large effective population size and low rates of genetic drift [2, 4]. However, it is noteworthy that there appears to be lower level of gene flow and population sub-structuring among *H. contortus* populations on a global scale, supported by the studies employing both microsatellite markers and the *nad*4 gene sequence data [9, 18]. Apparently, the restricted movement of livestock associated with the trade barriers and agricultural policies can lead to reduced gene flow and the formation of ‘isolated’ *H. contortus* populations.

## Conclusions

This is the first population genetic study of *H. contortus* in China using microsatellite markers. The findings provide a snapshot of the genetic make-up of *H. contortus* populations in parts of China using these polymorphic markers, revealing high within-population variation, low population genetic differentiation and high gene flow for *H. contortus*. This study should provide a reference for assessing genetic change in *H. contortus* over space and time during or following the implementation of particular treatment or control strategies, or as a consequence of environmental, management and climatic factors.

## Additional file

Additional file 1: Figure S1. Locations of seven populations of *Haemonchus contortus* in China. (TIF 2529 kb)

## Abbreviations

AMOVA: Analysis of molecular variance
*H*_*e*_: Expected heterozygosities
*H*_*o*_: Observed heterozygosities
HWE: Hardy-Weinberg equilibrium
IAM: Infinite alleles model
ITS-2: The second internal transcribed spacer of ribosomal DNA
PCoA: Principal coordinate analysis
PCR: Polymerase chain reaction
PIC: Polymorphism information content
SMM: Stepwise mutation model
TPM: Two-phase model
