# Supplementary figures and images for: Population structure of Haemonchus contortus from seven geographical regions in China, determined on the basis of microsatellite markers
Source: Parasit Vectors. 2016 Nov 15;9:586. doi: 10.1186/s13071-016-1864-z (PMC5111246; doi:10.1186/s13071-016-1864-z)

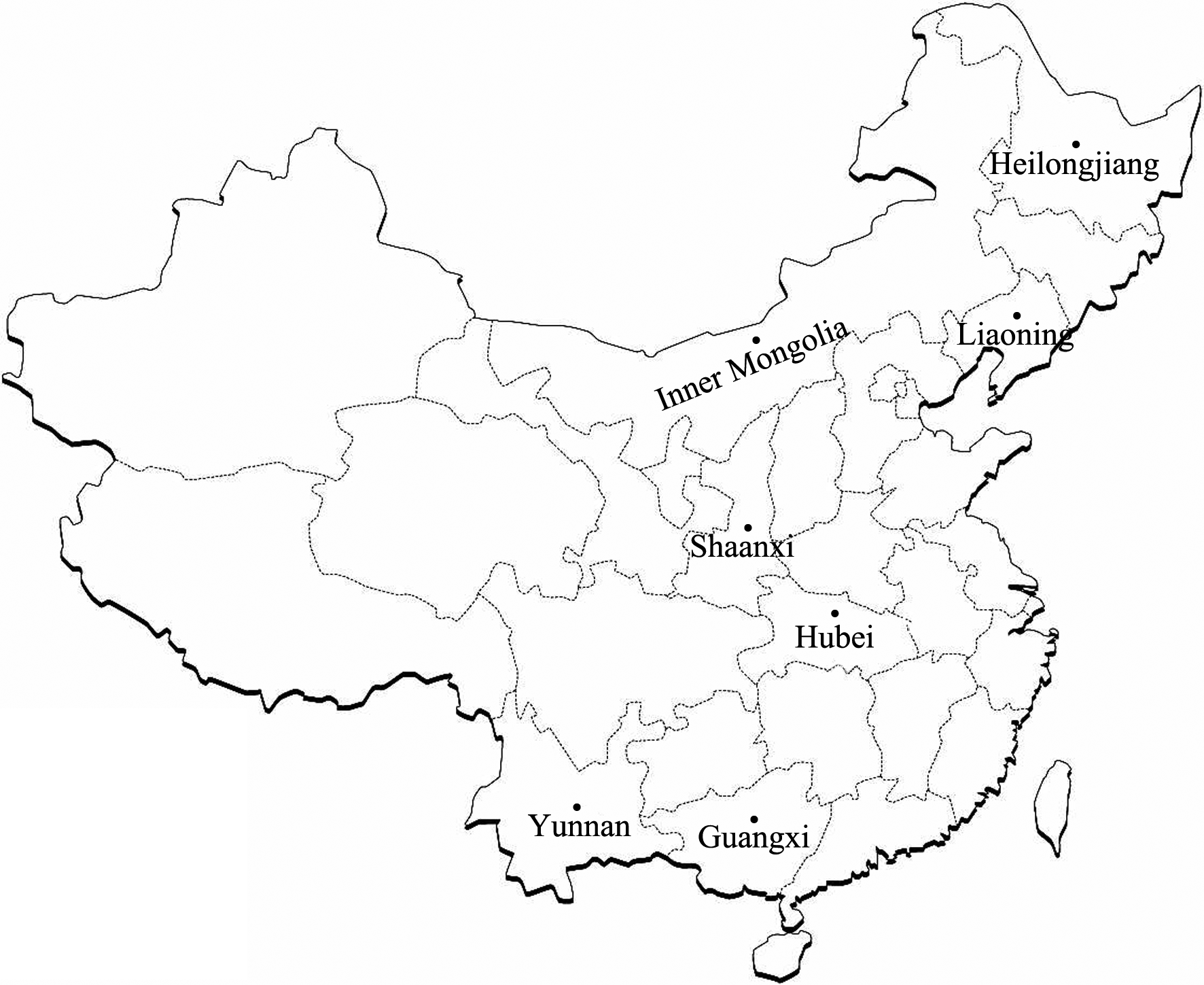

Supplement: Additional file 1: Figure S1. — Locations of seven populations of Haemonchus contortus in China. (TIF 2529 kb) [file 13071_2016_1864_MOESM1_ESM.tif]
